# Nonlinear multi independent variables in quantifying river bank erosion using Neural Network AutoRegressive eXogenous (NNARX) model

**DOI:** 10.1016/j.heliyon.2024.e26252

**Published:** 2024-02-16

**Authors:** Azlinda Saadon, Jazuri Abdullah, Ihsan Mohd Yassin, Nur Shazwani Muhammad, Junaidah Ariffin

**Affiliations:** a School of Civil Engineering, College of Engineering, Universiti Teknologi MARA, 40450, Shah Alam, Selangor, Malaysia; b Microwave Research Institute (MRI), Universiti Teknologi MARA, 40450, Shah Alam, Selangor, Malaysia; c Department of Civil Engineering, Faculty of Engineering and Built Environment, Universiti Kebangsaan Malaysia, 43600, Bangi, Selangor, Malaysia

**Keywords:** Natural river, NNARX, Riverbank erosion rate, Nonlinear behaviour, Sensitivity analysis

## Abstract

This study proposed a novel application of Neural Network AutoRegressive eXogenous (NNARX) model in predicting nonlinear behaviour of riverbank erosion rates which is difficult to be achieved with good accuracy using conventional approaches. This model can estimate complex river bank erosion rates with flow variations. The NNARX model analysed to a set of primary data, 60% (203 data for training) and 40% (135 data for testing), which were collected from Sg. Bernam, Malaysia. A set of nondimensional parameters, known as functional relationship, used as an input to the NNARX model has been established using the method of repeating variables. The One-Step-Ahead time series prediction plots are used to assess the accuracy of all developed models. Model no. 6 (5 independent variables with 10 hidden layers) gives good predictive performance, supported by the graphical analysis with discrepancy ratio of 94% and 90% for training and testing datasets. This finding is consistent with model accuracy result, where Model no. 6 achieved R^2^ of 0.932 and 0.788 for training and testing datasets, respectively. Result shows that bank erosion is maximized when the near-bank velocity between 0.2 and 0.5 m/s, and the riverbank erosion is between 1.5 and 1.8 m/year. On the other hand, higher velocities ranging from 0.8 to 1.3 m/s induces erosion at a rate between 0.1 and 0.4 m/year. Sensitivity analysis shows that the highest accuracy of 91% is given by the ratio of shear velocity to near-bank velocity followed by boundary shear stress to near-bank velocity ratio (88.5%) and critical shear stress to near-bank velocity ratio (88.2%). It is concluded that the developed model has accurately predicted non-linear behaviour of riverbank erosion rates with flow variations. The study's findings provide valuable insights in advanced simulations and predictions of channel migration, encompassing both lateral and vertical movements, the repercussions on the adjacent river corridor, assessing the extent of land degradation and in formulating plans for effective riverbank protection and management measures.

## 1 Introduction

Riverbank erosion rate estimation can be carried out in terms of space (spatial) or time (temporal) or the integration of both approaches [1]. Nevertheless, these two approaches require a broad range of time series data to forecast future data. Research on the development of empirical models to estimate bank erosion rate has evolved for many years. Because of this, the governing parameters of one model may differ from the other models depending on various factors including applied shear stress on the bank materials, bank slope, bank cover, cohesive strength of the materials that influence the bank and water level fluctuations. Various approaches such as empirical methods, numerical simulations, physical-based models, or integration of these methods, among others, have been employed in the estimation of sediment rates.

Mathematical models were also commonly used [[2], [3], [4], [5]]. However, extensive data requirements and rigorous model development are needed, which leads to a longer processing time. The development of practical mathematical model techniques, such as multi-linear regression (MLR), has been proposed by previous researchers for sediment modelling [6,7], riverbank erosion prediction [8,9], and channel migration prediction [[10], [11], [12]]. Nevertheless, this model focused on a linear relationship between the variables, which may not accurately represent the actual natural conditions of the study area. The actual natural condition subjected to the trends or seasonal weather variations that could lead to changes in water levels and flow velocity, especially during low and high flows, which impacted the stability of the banks. Theoretically, the rate of bank erosion is highly dependent on the incremental of flows, however, certain cases with regards to the bank stability, the erosion rates could yield a higher value at lower flowrates. This is due to the geometry of the riverbanks that signifies the bank stability. Given that scenario, linear approaches are unable to accurately imitate the highly non-linear natural behaviour of riverbank erosion as it involves interaction between several parameters, such as hydraulics, channel geometry and soil properties.

Problems related to nonlinear spatial and temporal values with high data dimensions can be comprehensively solved using Artificial Neural Network (ANN). The ANN allows machine learning to solve various problems related to prediction and classifications [6,[13], [14], [15]]. The advancement of machine learning and artificial intelligence, including the use of numerous methods related to artificial neural network (ANN), assists meticulous analysis to be faster and more efficient. The neural network uses the process of training input parameters and target that has been determined, to identify a reliable output pattern. With that ability, application of ANNs in erosion and sediment transport process has been explored by various research, including recent studies related to suspended sediment forecasting [[16], [17], [18], [19], [20]], sediment discharge prediction [21], bed load prediction [22,23], coastal erosion [24], soil and watershed erosion [25], and scour-depth prediction [26]. Through comprehensive literature review, the authors found that the application of ANN model in erosion and sediment transport (specifically suspended sediment, bed-load prediction, soil and watershed erosion, and coastal erosion) were limited to the above-mentioned studies. Studies related to riverbank erosion are very much lacking due to the challenges in obtaining field data, which are time consuming, expensive, weather-dependent, and complex soil-water interactions [2,25].

Other than finding the most suitable ANN model to forecast riverbank erosion rate of a natural river [1], have raised their concern on the need to examine the sensitivity of the chosen parameters in the empirical models to reduce errors arising from modelling concept and assumptions. Sensitivity analysis, especially in determining the model output uncertainty and its source is fundamental [27]. The main aim of a such analysis is to identify the most sensitive input parameters that causes changes in model output parameters [28]. Past research performed sensitivity analysis using various methods. These methods may be as straightforward as a simple one-factor-at-a-time method to more complex techniques such as Monte Carlo based method [[29], [30], [31]]. Numerous studies [[32], [33], [34], [35]] that examines the sensitivity of several erosion models, for example ANSWERS [36], GUEST [37], EUROSEM [38], USLE [39], PSEM-2D [40], MPSIAC [41], and SWAP [42]. In predicting flow, sediment, and nutrient discharge [43], performed sensitivity analysis using the Soil and Water Assessment Tool (SWAT) model. Their study included watershed characteristics such as watershed area, drainage density, average slope, and soil erodibility.

To accurately quantify riverbank erosion rates, river and sediment characteristics must also be considered. Although ANN has been used widely with good accuracy, problems related to erosion interacts closely to the dynamics of flow and fluctuation of water levels due to flood, which have been proven to be highly non-linear [44]. The common element behaviours of features in nonlinearity datasets enable the formation of auto-generated visual patterns [45], thus, classification or regression using linear or mildly non-linear functions is difficult to achieve [2]. This problem has been shown by the less successful model by Ref. [46], where the variables were linearized using principal component analysis and multi regression models. Due to this limitation, a more advanced Non-linear Auto Regressive model with eXogenous inputs (NARX) model is proposed to manage data highly scattered and varied data. NARX input models are used to solve continuous process regression problems that require all output variables to be presented in continuous or discrete-time sequences. Consequently, NARX can be combined with regression models, such as logistic regression to deal with multicollinearity [47]. Most of the developed models did not include model parameterization or sensitivity analysis as part of the parameter's evaluation of the model output. Several attempts were made [48,49] using NARX with additional QR factorization inputs (NARX-QR) to improve the accuracy of the prediction model with regards to riverbank erosion rate. The simulated NARX-QR model was dependent on the condition of regressor matrix and successfully generated R^2^ model prediction up to 90.6% accuracy. However, due to the nonlinearity of the datasets, the Neural Network NARX (NNARX) model can be used to further improve model accuracy. NNARX is one of the many estimators of NARX with known ability to overcome nonlinearity problems.

Therefore, this study focuses on estimating riverbank erosion rates, including during high and low flows, with a high accuracy in identifying the most significant factor that contributes to the erosion rate and establish a functional relationship between riverbank erosion rate and input parameters: namely hydraulics characteristics, riverbank geometry and soil properties. The developed model can be used to predict the rate of riverbank erosion subjected to both low and high flows. NNARX, a fast and rigorous artificial neural network model that allows multiple analyses of the parameters that includes the analysis of sensitivity of input parameters. Based on comprehensive literature review, the authors found that this is the first application of NNARX to predict erosion rates of the bank vertical surface, including sensitivity analysis of all input parameters. Further application of the established model can be linked to the analysis of riverbank stability, lateral migration, and meandering prediction.

## 2 Methodology

**Fig. 1:**
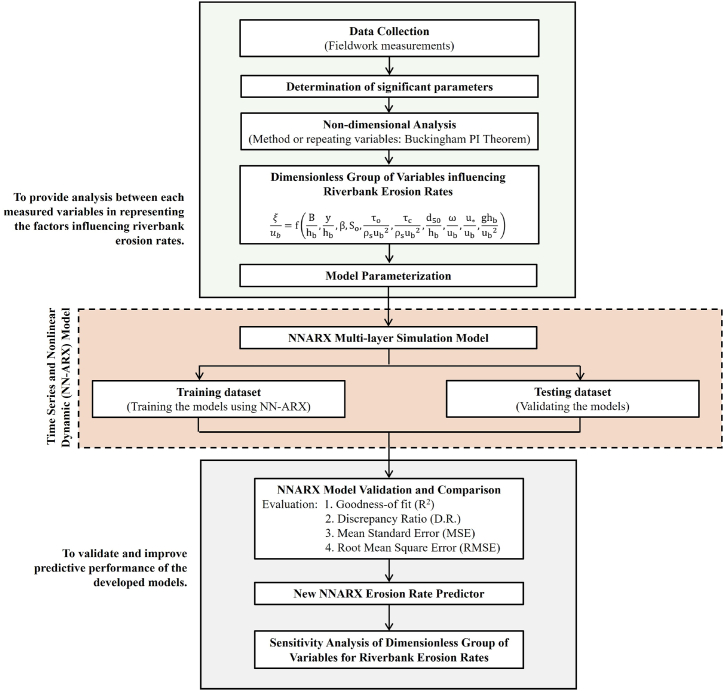
Flow chart of the methodology.

The methodology is shown graphically in Fig. 1, while detailed discussion on the methodology is given in the following sections.

### 2.1 Data collection

Sg. Bernam (3^o^51.02′ N, 100^o^50.15’ E) is in the state of Selangor, Malaysia with an area of slightly more than 2800 km^2^. The dataset used in this study were obtained from extensive fieldwork measurements at Sg. Bernam, Selangor, which include river hydraulics characteristics, riverbank geometry and riverbank erosion characteristics. Fieldwork measurement has been conducted for 12 consecutive months, comprising of both low and high flows.

Conventional erosion pins were used to measure riverbank erosion rates, a 60 cm metal rod length, 6 mm diameter (as suggested by Ref. [50]). 10 cm of the erosion pins were exposed and driven perpendicular to the riverbank face. Five pins were inserted at each interval plot for both left and right side of the selected banks. Measurements were taken by measuring the exposed length of each pin every two weeks and after each storm flow event. The amount of erosion perpendicular to the riverbank face are measured as erosion length in unit depth (mm), every two weeks. The average riverbank erosion rates (in mm/day) are estimated based on ratio of erosion depths and number of days.

**Fig. 2:**
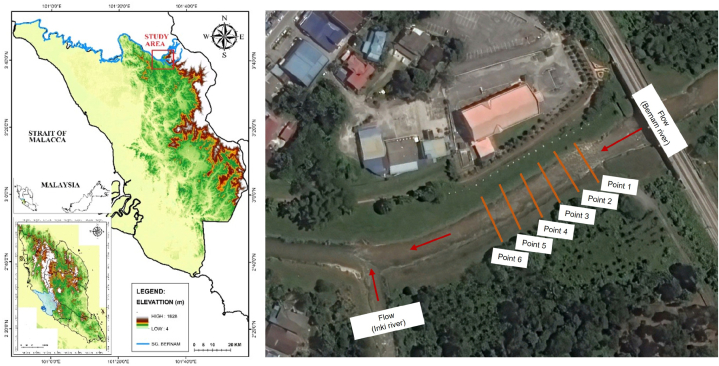
Map of the study area (Sg. Bernam at Tanjung Malim).

Due to the high frequency of fieldwork measurements in a year, the research team has successfully gathered a total of 338 observed samples. During observation, some pins were submerged underwater due to rise of water level after a storm event, of which the reading of pins was assisted using an underwater camera. Due to the deposition of bank materials especially on the outer bank, some pins were buried and were missing. They were replaced for future measurements. A 50 *m* stretch of straight channel was selected and it is equally discretised into 6 sections of 10 *m* interval, as shown in Fig. 2. Based on our observation, the reach has on-going occurrence of eroded bank. The bank height and bank angle ranging between 3.7 *m* and 3.8 *m* and 60–70°, respectively. River gauging exercise was performed using mean-section method and near-bank velocity is measured using the method proposed by Ref. [51].

**Fig. 3:**
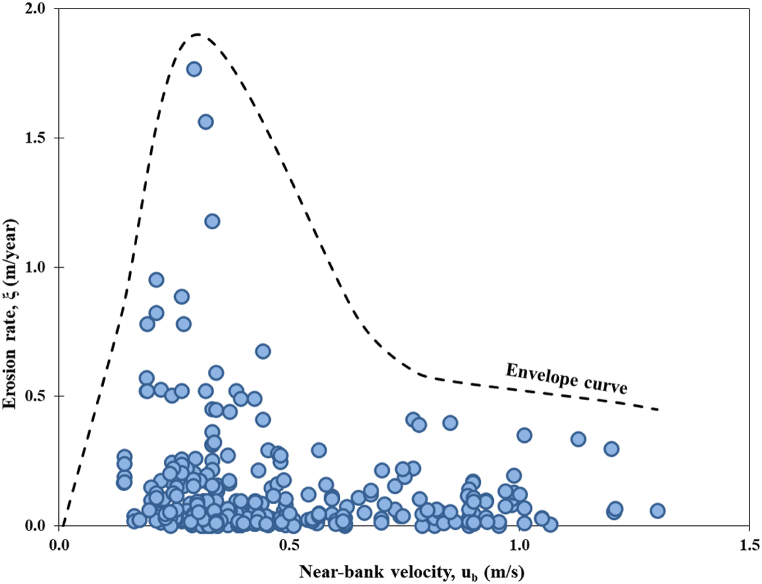
Erosion rate envelope curve for Sg. Bernam based on fieldwork measurements (modified from Ref. [2]).

The measured bank erosion rates can be represented in the form of an envelope curve, as shown in Fig. 3. This is also significant to the envelop curve developed in Refs. [52,53] representing the migration rate per year against the radius river bank curvature. Bank erosion rates, especially that considers both high and low flows is difficult to be quantified accurately. Theoretically, the rate of river bank erosion would ideally be directly proportional to changes in flow. However, in actual conditions, river bank erosion can also be influenced by fluctuations in water levels resulting from both high and low flow conditions [[54], [55], [56]]. In situations with a stable river water level, a river bank characterized by higher resistance force experiences failure with a larger and deeper overhang erosion width. Conversely, when the river water level rises, a less cohesive soil bank will erode over a broader width, resulting in river bank failure. Consequently, the rate of erosion is not solely dictated by flow rates but is also governed by other factors. These factors include bank geometry, the presence of tension cracks on the river bank that can reduce bank stability, variations in bank material particle sizes, the impact of pulses striking the bank, and the distribution of vegetative cover. It could be observed that the bank erosion is maximized between 1.5 and 1.8 m/year when the near-bank velocity between 0.2 and 0.5 m/s. On the other hand, higher velocities ranging from 0.8 to 1.3 m/s induces erosion at a rate between 0.1 and 0.4 m/year. Fieldwork observation indicated that the soil bank eroded mostly on the outer bank, and it is often a combination of under cutting due to variation of water levels during storm events. Low velocity at that location caused the eroded soil to be deposited on the inner bank and most heavier particles (sand) settle down while finer particles are carried away downstream to the sea or lake.

### 2.2 Determination of significant parameters to riverbank erosion

Nondimensional analysis utilizing the Buckingham PI theorem, also referred to as the π-theorem or method of repeating variables, and model parameterization was undertaken to meticulously assess the key parameters for the NNARX multi-layer simulation model. The PI refers to a set of nondimensional parameters generated through the method of repeating variables. These nondimensional parameters are examined to establish a functional relationship for quantifying river bank erosion rates, encompassing diverse input parameters, including hydraulic characteristics, riverbank geometry, and soil properties. The first stage utilized the method of repeating variables to establish a functional relationship between the DV and IVs. Following this, these dimensional variables were classified into three categories, namely bank geometry, hydraulic characteristics (flow resistance), and soil-grain characteristics, as shown in Table 1. It should be noted that these classifications were made to represent the main influencing factors of riverbank erosion rates. In model parameterization, each IV and DV were examined in terms of its significance and correlation based on the output of non-dimensional analysis. Previous researchers [39] concluded that the selection of IVs was made based on two major categories, namely migration parameters and riverbank erosion. The existing equations established by previous researchers suggested that erosion parameters are related to bed particle size, flow-induced forces, concentration of suspended sediment, bank particle size, channel gradient, channel width, vegetation protection and planform geometry. Dimensional analysis output is termed functional relationship, which represent the dimensionless parameters that signify to riverbank erosion.

**Table 1:** Dimensional variables and its categories.

Categories	Dimensional variables	Representation	Measurement Units
Bank geometry	Width of Channel	*B*	m
	Depth of water	*y*	m
	Height of riverbank	*h*_*b*_	m
	Angle of riverbank	*β*	–
	Bed slope of channel	*S*_*o*_	–
Hydraulic	Riverbank erosion rate	ξ	m/s
	Shear velocity	u*	m/s
	Near-bank velocity	*u*_*b*_	m/s
	Critical shear stress	*τ*_*c*_	N/ms^2^
	Boundary shear stress	*τ*_*o*_	N/ms^2^
Soil and grain characteristics	Bank particle density Mean particle diameter	*ρ*_*s*_*d*_*50*_ *ω*	kg/m^3^ m m/s
	Fall velocity		

For all established parameters, scatter plots DV against IV were developed and examined, with DV plotted on the vertical axis and each IVs plotted on the abscissa (horizontal axis). These dimensionless parameters were assessed using statistic correlation function, i.e., correlation coefficient (R^2^). Dimensionless parameters with high coefficient correlation (R^2^) greater than 0.5 is adequate but values closer to 1.0 is recommended [2,49]. Additionally, parameters with high correlation values (positively correlated or negatively correlated) may be significant and worthy of attention for further analysis.

### 2.3 Neural network nonlinear AutoRegressive model with eXogenous (NNARX) inputs model structure

#### 2.3.1 Input data

The controlling parameters for riverbank erosion rates derived from non-dimensional analysis are used as input data to the NNARX model. The input parameters include the DV and IV that represent significant riverbank erosion factors. The proposed NNARX model requires the dataset to be arranged in time-series order. Therefore, 338 erosion pins are used during the field measurements as mentioned in the previous section were fed into the NNARX model. The input data for the model uses the optimal split proportions. The split sample approach is a widely employed technique for studying design in high-dimensional settings [2,57,58]. This approach is founded on simulations aimed at qualitatively understanding the relationships among dataset characteristics, particularly data sizes ranging from 100 to 1,000,000. In this methodology, the data is partitioned into training and testing sets [57]. suggested an allocation of between 1/2 and 3/4 of the data for training, with the remainder dedicated to testing. Consequently, in this study, 60% (203 data points) of the data were used for training, and the remaining 40% (135 data points) were reserved for testing the empirical model development. The optimal proportion was found to be contingent on the full dataset size (n) and the classification accuracy, where higher accuracy and smaller dataset size (n) resulted in a larger portion being assigned to the training set. The common practice of allocating two-thirds of the overall data for training was found to be close to optimal for reasonably sized datasets (n > 100). In the developed NNARX model, the target outputs were the measured riverbank erosion rates.

#### 2.3.2 NNARX modelling

NNARX model is a multi-layer Neural Network (NN) model incorporating of Non-linear Auto Regressive eXogenous (NARX) inputs to predict the riverbank erosion rate. This model has been chosen based on its robustness in handling data that are extremely non-linear and data with diverse characteristics, uncertainties, and unknown disturbance.

In this study, series-parallel (open loop) architecture was selected for training stage based on the chronological order of historical (observed field measurements) time series. The designed network architecture offers two major advantages, i.e., input parameters, i.e., the observed field measurements are used in the feedforward network, and therefore, the Multi-Layer Perceptron (MLP) algorithms, that is usually used for training can be used.

There are three layers in a conventional MLP, namely input, hidden and output. These layers are interconnected using weighted connections and are activated by activation function that fire when sufficiently strong inputs are received. Each neuron in every layer multiplies the input vector (*x*_*i*_) provided by previous layer and weights vector (*w*_*ij*_) to produce the scalar product (*x*_*j*_*∙w*_*ij*_). An activation function, *f*, is estimated using Equation (1).

(1) $$y_{i}=f\left(\sum_{j=1}^{n}x_{i}.w_{ij}\right)$$

where *i* is the neuron index in the layer and *j* is index of input. Additionally, bias connections were also added to improve network convergence.

**Fig. 4:**
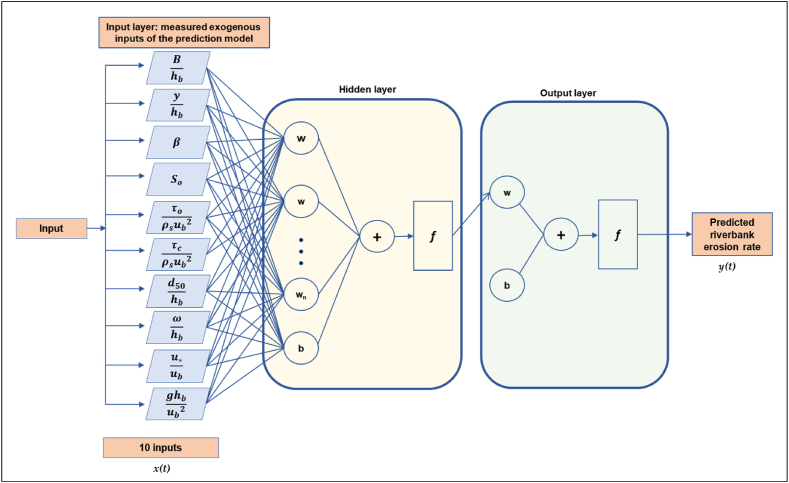
Details of recurrent neural network (neural network closed-loop architecture) for NNARX riverbank erosion prediction model.

Fig. 4 comprehensively shows the variables used, number of neurons and the recurrent neural network architecture used in the learning and training of the data, known as the NNARX riverbank erosion prediction model. A supervised learning and training process modifies the set of weights using a suitable algorithm (backpropagation or its numerical variants such as the Levenberg-Marquardt or Scaled Conjugate Gradient), to ensure that it is done properly. Specifically, a certain number of input data and the desired output (target) are assigned to the NNARX model. Variations of weights are assigned to make sure that the model can produce the outputs that are close to the target values. However, it should be noted that any NNARX model typically has error or residuals (i.e., “noise”), because knowledge of the other terms does not facilitate in the prediction of the current time series values. The predictor, derived from the NNARX model known as *y(t)*, is a black box type nonlinear function.

The choice of variables, type of activation function in the hidden layer, number of neurons used, and the training weights determine the accuracy of the predictive model.

The tangent-sigmoid function was employed as the activation function in the hidden neurons and bias connections. The number of hidden units were varied (either 10, 15 or 24). The choice of hidden units is arbitrary but has significant effect on the results. Typically, we want the selection to obey the principle of parsimony, where we want the minimum number of units to achieve the best outcome. The tangent-sigmoid activation function is like the sigmoid function albeit less computationally expensive. The activation function tapers off at −1 and +1 with an approximately linear curve connecting the two extremes. This is designed to generate a strong unit output (firing either positive or negative valued) if its weighted inputs are strong enough.

**Fig. 5:**
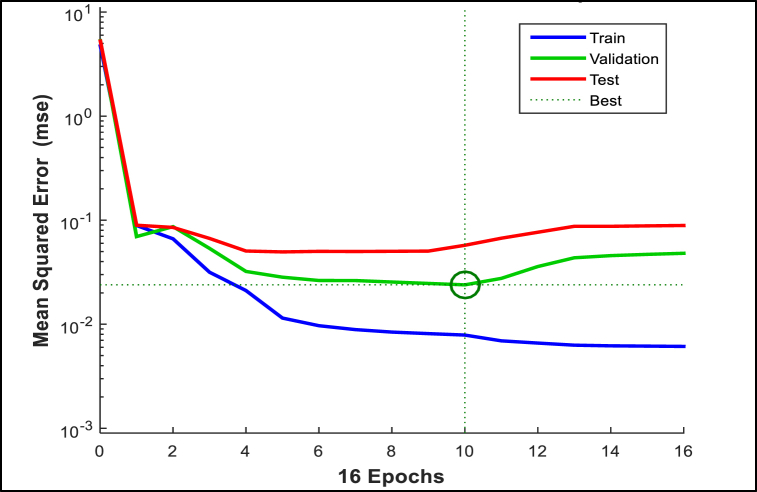
Graph of MSE versus weight adjustment value (early stopping).

Random weights were applied initially using the Nguyen-Widrow algorithm. The Nguyen-Widrow algorithm positions the random weights near the middle of the activation function. This allows the weights to be more freely adjusted during training (leading to fast training convergence) compared to the weights being placed closer to the maximum and minimum locations in the activation function. During training, the weights are adjusted to obtain the desired output (target) and simultaneously minimizing errors (difference between the predicted and target output). The early stopping algorithm was introduced in the NNARX model (as shown in Fig. 5) to avoid the weights from being over-adjusted to “memorize” the training data (an undesirable condition commonly known as overfitting). The early stopping algorithm works by “sensing” when overfitting nearly occurs (with the aid of an independent validation dataset) and stopping training just before overfitting occurs.

### 2.4 Model evaluation and comparison

The output (target) is temporal erosion rates which is defined as the riverbank erosion rates (*ξ*_*b*_) to the near-bank velocity (*u*_*b*_.) ratio. The NNARX model was responsible for prediction of the output in numerical form. To evaluate the predictive model capability, One-Step-Ahead (OSA) plots were generated based on the erosion rate against time. The OSA examines the model capability by using past data to predict the one-step-ahead output. Other than that, several statistical index-based parameters such as goodness-of-fit of the model (R^2^), Mean Square Error (MSE), Root Mean Square Error (RMSE) and Discrepancy Ratio (DR) were used to evaluate and compare the performance of the developed models [2,7,48,59].

Prior to model evaluation, the established erosion rate predictor was tested using sensitivity analysis to examine the relationship of each dimensionless groups of IV and DV derived from the NNARX model output. Observation of the scatter plot (DV against IV) trends were examined for all established functional relationships. A forty-five-degree inclination in the trends indicate some correlation exist between the DV and the dimensionless groups. This should be consistent with the statistical parameters that were used in model evaluation and comparison.

## 3 Results and discussion

Dimensional analysis was performed to determine the repetition of independent variables (IV) as inputs to the NNARX model. Functional relationships established from the dimensional analysis consisted of dimensionless parameters affecting the riverbank erosion rate. These parameters, known as IV to riverbank erosion rate, were selected based on the trends of scatter plot between each IV to the DV, as presented in Section 2.2. The controlling parameters for riverbank erosion rate obtained from dimensional analysis are also known as the input variables to the NNARX model. The accuracy of the established parameters used in the NNARX was evaluated using R^2^ and further validated using DR, MSE and RMSE.

### 3.1 Dimensional analysis

**Fig. 6:**
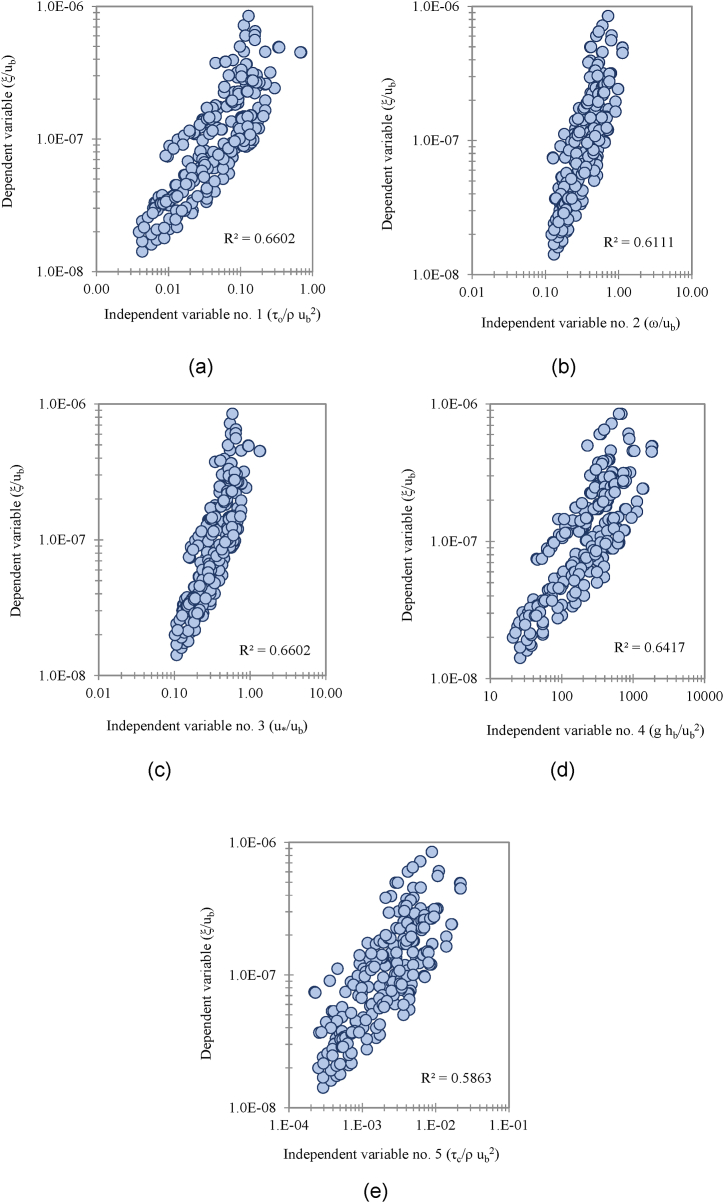
Trend of scatter plots between each IV (a*) τ*_*o*_*/ρ*_*w*_*u*_*b*_^*2*^; (b) *ω/u*_*b*_^*2*^; (c) u*/u_*b*_; (d) *g h*_*b*_*/u*_*b*_^*2*^; and (e) *τ*_*c*_/*ρ*_*w*_*u*_*b*_^*2*^ to DV, *ξ/u*_*b*_.

The relationship between the IV to the DV is a function of 14 variables as shown in Equation (2). Using the method of repeating variables, 11 dimensionless groups were generated based on three selected parameters as repeating variables (Equation (3)). Repeating variables must be selected wisely as it has the potential to appear in each number of expressions. This is to ensure the accuracy and significance relationship between IV and DV. From this analysis, 11 dimensionless parameters were established. The repeating variables selected are riverbank height, $h_{b}$, near-bank velocity, $u_{b}$, and sediment mass density, $\rho_{s}$. The final functional relationship after the Buckingham PItheoremanalysisyieldstheexpressionslistedinEquation3. The next stage is to evaluate the correlation between the dimensionless parameters using sensitivity analysis. The DV parameter were plotted against each IVs, the trends show positive sign on the important variables for development of the model. Five of the IV demonstrate positive relationship with the DV. Fig. 6(a) to Fig. 6(e) show trends of five significant parameters with positive correlation with the dependent variable.

(2) $$\xi =f\;\left(B,Y,h_{b},\beta ,S_{o},u_{b},\tau_{o},\tau_{c},d_{50},\omega ,u_{*},g,\rho_{s}\right)$$

(3) $$\frac{\xi }{u_{b}}=f\left(\frac{B}{h_{b}},\frac{Y}{h_{b}},\beta ,S_{o},\frac{\tau_{o}}{\rho_{s}{u_{b}}^{2}},\frac{\tau_{c}}{\rho_{s}{u_{b}}^{2}},\frac{d_{50}}{h_{b}},\frac{\omega }{u_{b}},\frac{\;u_{*}}{u_{b}},\frac{gh_{b}}{{u_{b}}^{2}}\right)$$

### 3.2 NN-ARX model

The NNARX model generated predicted values for the riverbank erosion rates as the output target. These values were compared with the observed fieldwork measurements as discussed in Section 2.1. Recall that the accuracy of the developed NNARX model was assessed using OSA plot and statistical parameters highlighted in Section 2.4. Based on the functional relationship derived in Section 3.1, six (6) configurations were tested in the NNARX network used in this study by interchanging the set of input parameters (consist of several IVs combinations) and adjusting the number of hidden layers. The selections of these IV were made based on the results from the model parameterization.

Model no. 1 consist of 11 IVs with 24 hidden layers applied to the NNARX input. Similar 11 IVs were used for a different set of hidden layers (Model no. 2 and Model no. 3, with 15 and 10 hidden layers, respectively). These units of hidden layers are responsible for the MLP's learning process by adjusting the weights between the layers. Based on the three configurations, ten unit of hidden layers managed to produce optimal results. The optimal hidden layers can be observed from the OSA prediction plots generated for all six model configurations. Ten hidden units were used in the following model development by reducing the number of IV to 9 (Model no. 4), 7 (Model no. 5) and 5 (Model no. 6).

**Fig. 7:**
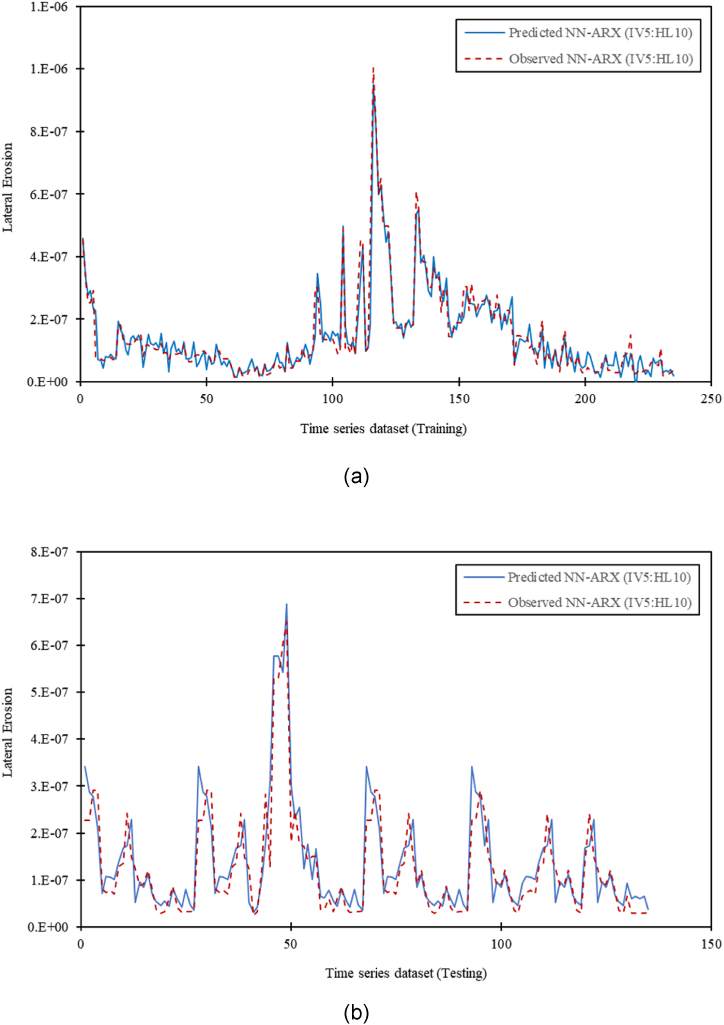
Convergence series based on OSA prediction plot between predicted erosion rates with measured erosion rates for (a) Model 6 (5IV, HL10) for training data; and (b) Model 6 (5IV, HL10) for testing data.

Model configurations tested and OSA prediction plots for both training and testing dataset for all six models were generated based on time series erosion rate. All six OSA prediction plots were examined, and predicted dataset resembled closely to the actual dataset are deemed to be accurate. Both training and testing OSA prediction plots for all six model configurations show good agreement between the NN-ARX model prediction (blue line) and observed data (red line), indicating good predictive performance. Fig. 7(a) and (b) show an example of the convergence series based on OSA forecast plots between predicted erosion rates with measured erosion rates for both training and testing dataset for Model no 6. It is evidenced that the NNARX input model with this configuration (5 IVs with 10 hidden layer) able to predict the measured dataset accurately. To further test the accuracy of the developed models, the remaining independent dataset were utilized in the model performance and validation.

### 3.3 Model validation and comparison

The summary of model performance and validation with different number of IV and hidden layers used in the NNARX model is presented in Table 2. In general, all six developed models using NNARX inputs exhibited good model performance, exhibiting percentage of successful prediction of riverbank erosion rates of more than 60% for both training and testing dataset. It is also evidenced that the MSE and RMSE calculated for all six models indicate a good model fit (MSE is small values and approaching zero). Additionally, the total percentage of the data lies within the DR limits (0.5–2.0) were calculated and visual plots using 1:1 graph between the predicted values and observed values were established.

**Table 2:** Summary of model performance and validation with different number of independent variables and hidden layers used in the NN-ARX model.

Input Parameters (Independent Variables)	Model no.	Number of Hidden Layer (HL)	Data set	Model Performance and Validation			
				DR	MSE	RMSE	R^2^
11	1	24	Training	84%	9.46 × 10^−13^	6.34 × 10^−8^	0.838
			Testing	74%	3.82 × 10^−13^	8.74 × 10^−8^	0.641
	2	15	Training	84%	1.78 × 10^−13^	2.75 × 10^−8^	0.952
			Testing	64%	1.03 × 10^−12^	1.43 × 10^−7^	0.255
	3	10	Training	92%	2.16 × 10^−13^	3.04 × 10^−8^	0.966
			Testing	80%	2.73 × 10^−13^	7.39 × 10^−8^	0.524
9	4	10	Training	91%	3.66 × 10^−13^	3.95 × 10^−8^	0.943
			Testing	86%	6.65 × 10^−13^	1.15 × 10^−7^	0.326
7	5	10	Training	82%	1.49 × 10^−12^	7.97 × 10^−8^	0.732
			Testing	50%	7.42 × 10^−13^	1.22 × 10^−7^	0.166
5	6	10	Training	94%	3.28 × 10^−13^	3.74 × 10^−8^	0.932
			Testing	90%	4.29 × 10^−13^	9.27 × 10^−8^	0.788

**Fig. 8:**
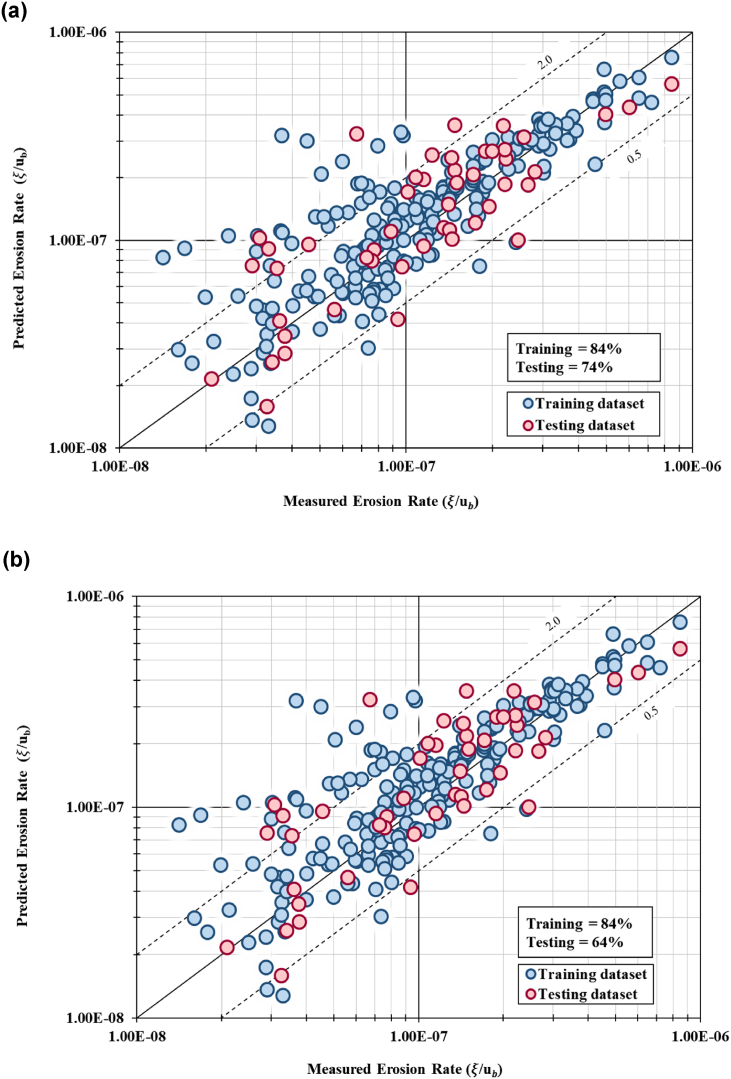

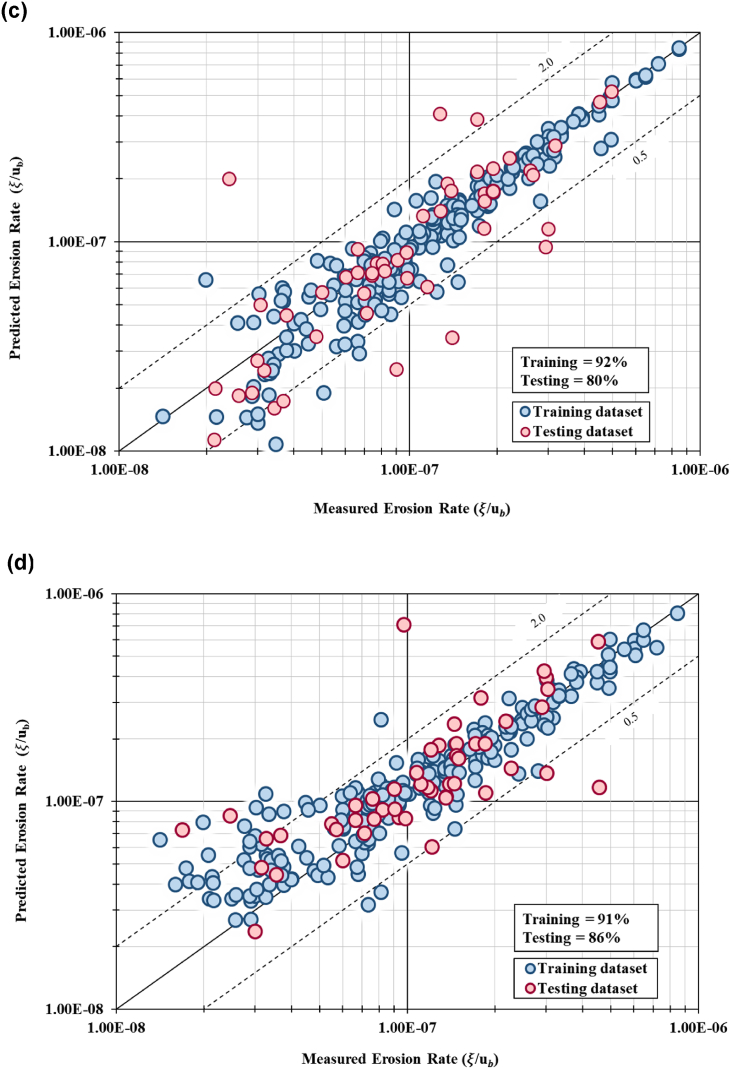

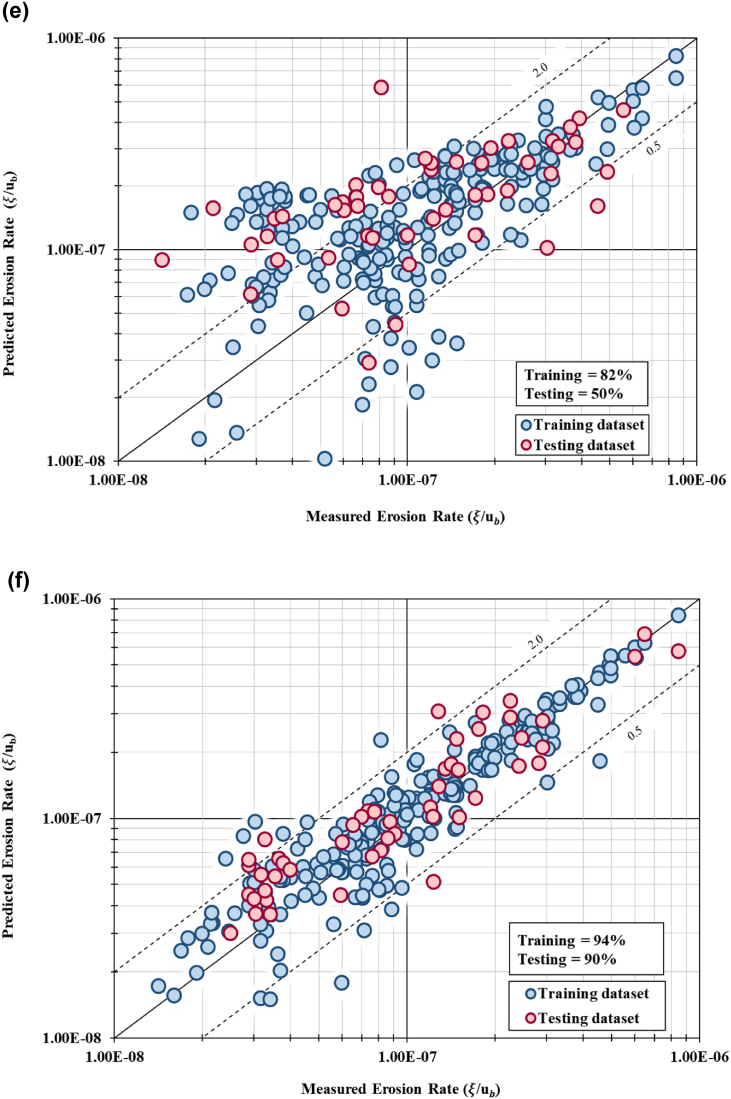
(a). 1:1 graph of predicted versus measured erosion rates for Model 1 (11 IV with 24 hidden layer) Fig. 8(b). 1:1 graph of predicted versus measured erosion rates for Model 2 (11 IV with 15 hidden layer) Fig. 8(c). 1:1 graph of predicted versus measured erosion rates for Model 3 (11 IV with 10 hidden layer) Fig. 8(d). 1:1 graph of predicted versus measured erosion rates for Model 4 (9 IV with 10 hidden layer) Fig. 8(e). 1:1 graph of predicted versus measured erosion rates for Model 5 (7 IV with 10 hidden layer) Fig. 8(f). 1:1 graph of predicted versus measured erosion rates for Model 6 (5 IV with 10 hidden layer).

Model validation confirmed that Model no. 6 using five independent variables with ten hidden layers achieved the highest percentage, where 94% of the training dataset lies between the limit of discrepancy ratio. The testing dataset for this model configuration achieved 90% of accuracy in the model validation. The MSE for this model indicates lowest value 3.28 × 10^−13^ for training dataset and 4.29 × 10^−13^ for testing dataset. The RMSE for this model depicts the lowest value of error at 3.74 × 10^−8^ for training dataset and 9.27 × 10^−8^ for testing dataset. This result is concurrent with the result from model accuracy where Model 6 achieved model goodness of fit (R^2^) of 0.932 for training dataset and 0.788 for testing dataset. All six prediction models achieved goodness-of-fit between 0.732 (73.2%) to 0.966 (96.6%). Model no. 1 (with 11 independent variables and 24 hidden units) achieved model fit of 0.838 (83.8%). It can be concluded that Model no. 3 with 10 hidden units predicted the highest goodness-of-fit value of 0.966 (96.6%) for training set and 0.524 (52.4%) fit for testing set. Model no. 6 with hidden units of 10 predicted accuracy at 0.932 (93.2%) and 0.788 (78.8%) for training and testing dataset, respectively. Fig. 8(a) to Fig. 8(e) show the graphical plot of measured values against predicted values of erosion rates using 1:1 graph. Model no. 1, 2, 3, 4 and 5 achieved high percentage for the validation (DR between 60% and 90%), however, the graphical analysis of Model no. 6 (i.e., Fig. 8(f)) produced a better trend with data scattered close to the regression line.

### 3.4 Sensitivity analysis

**Fig. 9:**
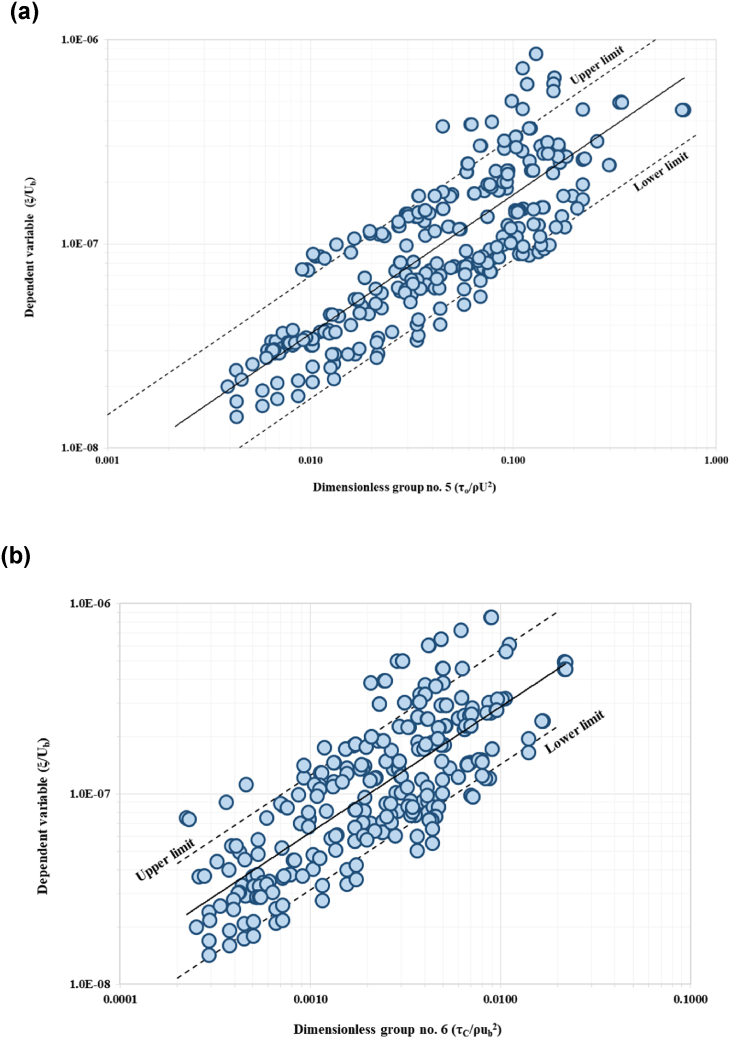

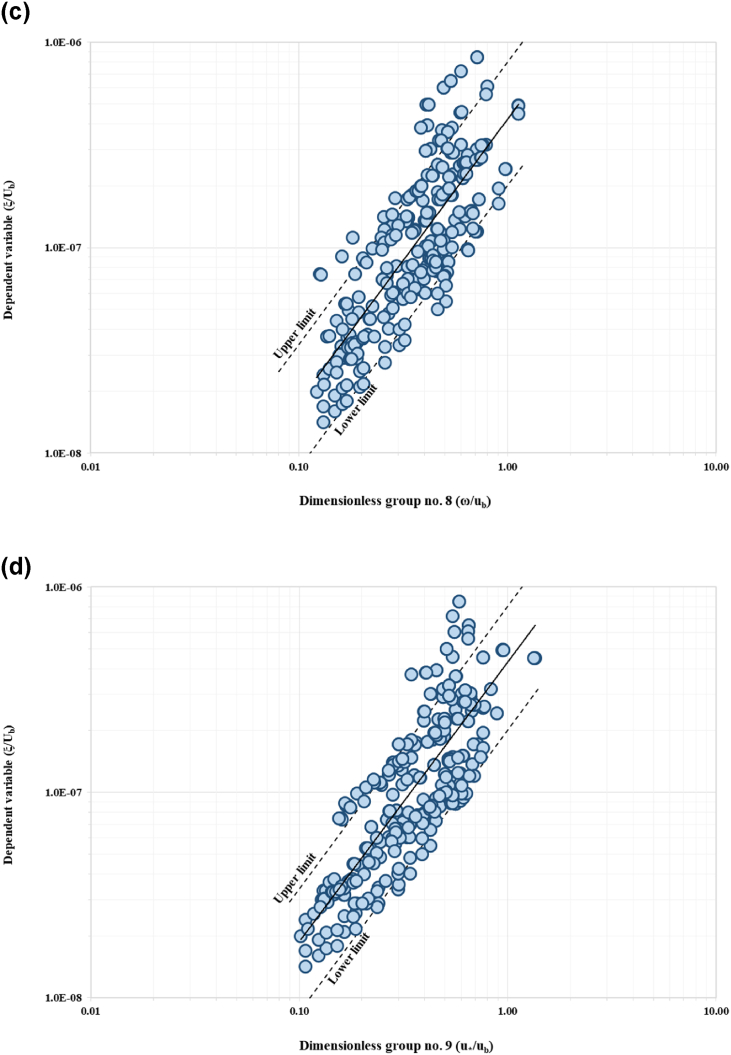

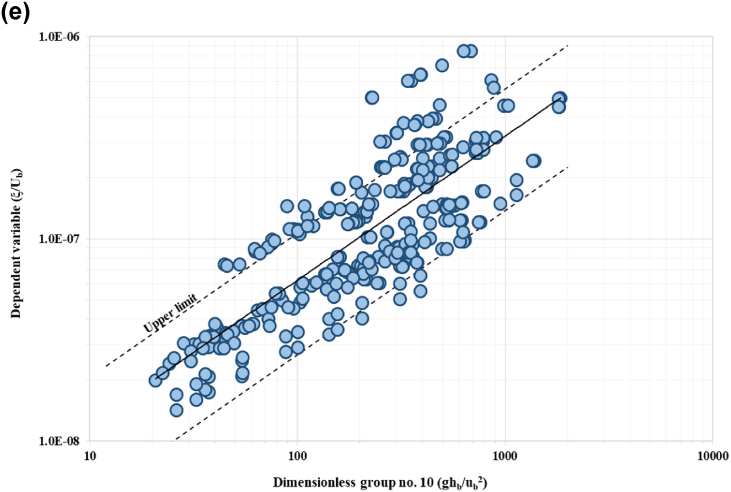
(a). Scatter plot of significant IV to the dependent variable with confidence boundary for boundary shear stress to near-bank velocity and sediment mass density (*τ*_*o*_/*ρ*_*s*_*u*_*b*_^*2*^) ratio. Fig. 9(b). Scatter plot of significant IV to the dependent variable with confidence boundary for the critical shear stress to near-bank velocity ratio and sediment mass density (*τ*_*c*_/*ρ*_*s*_*u*_*b*_^*2*^). Fig. 9(c). Scatter plot of significant IV to the dependent variable with confidence boundary for the fall velocity to near-bank velocity (*ω/u*_*b*_) ratio. Fig. 9(d). Scatter plot of significant IV to the dependent variable with confidence boundary for shear velocity to the near-bank velocity (u*/*u*_*b*_) ratio. Fig. 9(e). Scatter plot of significant IV to the dependent variable with confidence boundary for bank height to near-bank velocity (*gh*_*b*_*/u*_*b*_) ratio.

For all developed models, the ratio of boundary shear stress over near-bank velocity and sediment mass density (*τ*_*o*_/*ρ*_*s*_*u*_*b*_^*2*^), the ratio of critical shear stress over near-bank velocity and sediment mass density (*τ*_*c*_/*ρ*_*s*_*u*_*b*_^*2*^), the ratio of fall velocity over near-bank velocity (*ω/u*_*b*_), shear velocity over near-bank velocity (*u* */*u*_*b*_) ratio and ratio of bank height to the near-bank velocity (*gh*_*b*_*/u*_*b*_) yield high correlation coefficient (R^2^) values of more than 0.5. These parameters signify strong correlation between the dimensionless group and the dependent variable (DV). The results from sensitivity analysis are summarised in Table 3, with Fig. 9(a) to Fig. 9(e) present the scatter plots of all significant dimensionless group to the DV, including the correlation confidence boundaries. These confidence bounds are subjected to the minimum and maximum bound following to the limit of DR highlighted in Section 2.4. Data lies within these minimum and maximum bound were represented in the form of correlation percentage. More than 80% of the plotted points coincided within the limit. Data from the dimensionless group are plotted on the abscissa (x-axis) versus dependent variable in the vertical axis (y-axis). Dimensionless group no. 9 (as shown in Fig. 9(d)) yields the best accuracy with approximately 90.5% of the data within the confidence bound. Also, the dimensionless group no. 5 (ratio of boundary shear stress to the near-bank velocity ratio and sediment mass density, *τ*_*o*_/*ρ*_*s*_*u*_*b*_^*2*^), shows 88.5% of the data falls within the confidence bound as represented in Fig. 9(a). The third highest dimensionless group was the sixth (critical shear stress to the near-bank velocity ratio and sediment mass density, *τ*_*c*_/*ρ*_*s*_*u*_*b*_^*2*^) exhibiting 84.6% accuracy as in Fig. 9(b). The ratio between fall velocity to the near-bank velocity, *ω/u*_*b*_ (dimensionless group no. 8) gives 88.2% accuracy. The ratio between height of the riverbank to the near-bank velocity (*gh*_*b*_*/u*_*b*_) yielded accuracy at 85.8%. The dimensionless group no. 9, i.e., ratio of shear velocity to the near-bank velocity (*u* */*u*_*b*_) yields the most influential parameter to the DV (ratio of erosion rate to the near-bank velocity, *ξ*_*b*_*/u*_*b*_). These two parameters were mutually associated in a strong positive correlation. Another notable finding was that the erosion rate increases symmetrically to the shear velocity of the flow by simply ignoring the denominator term of the parameter's fraction.

**Table 3:** Overall results of sensitivity analysis for each dimensionless group, dimensionless parameters, and the correlation coefficient (R^2^).

$\frac{\xi }{u_{b}}=f\left(\frac{B}{h_{b}},\frac{Y}{h_{b}},\beta ,S_{o},\frac{\tau_{o}}{\rho_{s}{u_{b}}^{2}},\frac{\tau_{c}}{\rho_{s}{u_{b}}^{2}},\frac{d_{50}}{h_{b}},\frac{\omega }{u_{b}},\frac{\;U_{*}}{u_{b}},\frac{gh_{b}}{{u_{b}}^{2}},\frac{\rho_{w}}{\rho_{s}}\right)$				
Dimensionless group	Dimensionless parameters	Correlation coefficient (R^2^)	Percentage of data (%)	
			Within the confidence bound	Out to the confidence bound
1	$\frac{B}{h_{b}}$	0.064	Insignificant	
2	$\frac{Y}{h_{b}}$	0.017	Insignificant	
3	β	0.005	Insignificant	
4	$S_{o}$	0.041	Insignificant	
5	$\frac{\tau_{o}}{{\rho_{s}u_{b}}^{2}}$	0.660	88.5	11.5
6	$\frac{\tau_{c}}{{\rho_{s}u_{b}}^{2}}$	0.586 (Significant)	84.6	15.4
7	$\frac{d_{50}}{h_{b}}$	0.004	Insignificant	
8	$\frac{\omega }{u_{b}}$	0.611	88.2	11.8
9	$\frac{U_{*}}{u_{b}}$	0.660 (Significant)	90.5	9.5
10	$\frac{{gh}_{b}}{{u_{b}}^{2}}$	0.641	85.8	11.2

This study established that the rate of riverbank erosion is characterized by the shear velocity variation. This trend was also observed for dimensionless group no. 8 highlighted in Fig. 9(c), that represents the ratio of fall velocity to the near-bank velocity (*ω/u*_*b*_). Dimensionless group no. 5 (boundary shear stress to the near-bank velocity and sediment mass density (*τ*_*o*_/*ρ*_*s*_*u*_*b*_^*2*^) suggests a significant correlation to the DV with 88.5% accuracy. The force per unit area exerted on the surface beneath and in the direction of the flow is known as the boundary shear stress, *τ*_*o*_. In other words, it corresponds to the forces of lift and drag utilized by the turbulent flows in objects protruding from the boundary in this study, which refers to the soil particles on the riverbank. This study estimates the boundary shear stress based on the mean soil particle size and water depth, as suggested by Ref. [60]. The rate of riverbank erosion varies, and this was possible due to the range of particle size resting on the river bank surface and water depth, as shown in the scatter plots. This finding is further verified by observing the trends for dimensionless group no. 6, i.e., critical shear stress to the near-bank velocity ratio. Dimensionless group no. 10 (Fig. 9(d)), height of riverbank to the near-bank velocity ratio (*gh*_*b*_*/u*_*b*_) yields a strong positive correlation with DV at 85.8%. The range of riverbank erosion rate increases simultaneously to the incremental of the riverbank characteristics, as shown in the scatter plot. Theoretically, riverbank height, especially steep banks approaching 90^o^ angle is prone to bank toe scouring, bank instability and bank failure. Field observation indicates that these banks with evidenced of bank toe scouring initiates mass bank failure and block failure. Another novel finding that needs to be highlighted here is that the sensitivity analysis has successfully derived and hence identify the most influential parameters in estimating the riverbank erosion rate.

## 4 Conclusion

An intelligent control-based system identification approach known as the NNARX was used to evaluate and establish riverbank erosion predictor inclusive of reach-scale hydraulic and bank-scale erosion nonlinear multi-independent variables. This study concluded that:

a) The first application of NNARX that accurately predicts the non-linear behaviour of erosion rates on a vertical bank surface, comprising of both low flow and high flow conditions. Input parameters, i.e., hydraulics characteristics, soil parameters and bank geometry of a natural river went through a rigorous model parameterization and sensitivity analysis. Eleven IV were derived as functional relationships using method of repeating variables. These parameters indicate the significant factors to streambank erosion rates and can be utilized for riverbank erosion predictor. These influential factors will be useful for the bank stability analysis that will link to the lateral migration prediction.
b) Six models successfully generated from NNARX model prediction with variation configuration of independent variables and hidden layers. Based on all six model configurations, ten units of hidden layers managed to produce optimal results. All models exhibited good standing, with the successful prediction of riverbank erosion rates with accuracies more than 60% for both training and testing datasets. On the same note, the OSA prediction plots for all six model configurations and both datasets were examined, and it can be concluded that all six models show good agreement between the prediction and actual datasets. Model no. 6 (5 IVs with 10 hidden layers) able to predict the actual dataset accurately.
c) Model validation confirmed that Model no. 6 consisting of five independent variables and 10 hidden units achieved the highest accuracy, having 94% and 90% of the training and testing datasets within the DR limit. This finding is consistent with model accuracy result, where Model no. 6 achieved R^2^ of 0.932 and 0.788 for training and testing datasets, respectively. The MSE for this model yielded lowest value for training and testing dataset. The RMSE for this model also yielded lowest value of error at 3.74 × 10^−8^ for training data set and 9.27 × 10^−8^ for testing dataset. It should be noted that Models no. 1, 2, 3, 4 and 5 also achieved high percentage of accuracy for the validation. However, the graphical analysis of Model no. 6 shows a better trend with all data scattered closely to the regression line. Hence, it can be concluded that Model no. 6 with 5 independent variables with 10 hidden layers best to represent the riverbank reach-scale hydraulic and bank-scale erosion predictor, hence, performed better than the other developed models.
d) Eleven IVs were considered for the model prediction. However, based on the sensitivity analysis in the model testing, only five dimensionless groups exhibited the most influential parameter to the dependent variable. Dimensionless group no. 9, *u* */*u*_*b*_ produced the best accuracy with approximately 90.5%, followed by the dimensionless group no. 5, *τ*_*o*_/*ρ*_*s*_*u*_*b*_^*2*^ with 88.5% accuracy, the *ω/u*_*b*_ yielded 88.2% accuracy, the *gh*_*b*_*/u*_*b*_ yielded 85.8% accuracy, and the *τ*_*c*_/*ρ*_*s*_*u*_*b*_^*2*^ shows 84.6% accuracy. These variables are also identified as the most significant parameters, with R^2^ value of more than 0.5.
e) Fieldwork data of bank erosion rate used in the NNARX model covering both high and low flows combination of undercutting due to variation of water levels during storm events, and governed by other aspects, such as the geometry of the bank, the existence of tension cracks on the river bank face that could reduce the stability of the river bank, variation of bank material particle sizes, the variation of pulses striking on to the bank, and distribution of the vegetative cover. It is observed that the bank erosion is maximized when the near-bank velocity between 0.2 and 0.5 m/s, and the riverbank erosion rate is 1.5–1.8 m/year. On the other hand, higher velocities ranging from 0.8 to 1.3 m/s induces erosion at a rate between 0.1 and 0.4 m/year. With good model performance, the established NNARX can be used for both low and high flows in a different river environment.
f) The developed model can be reproduced, replicated, and tested in another river that has similar characteristics as these derived parameters, since input data are mainly nondimensional parameters.

The findings of the study will be helpful in controlling river bank erosion and assist in formulating proper strategy in a sustainable way through various aspects. Riverbank erosion prediction models play a crucial role in quantifying the extent of erosion and identifying areas prone to riverbank erosion. These models consider multiple factors, including hydraulic characteristics, bank-soil parameters, vegetative coverage, bank geometry, and various other variables. The predictions generated by these models are valuable for planning riverbank protection and mitigation strategies that align with sustainability principles. Such strategies may encompass soil-bioengineering riverbank protection measures, such as root reinforcement in the soil, vegetated riprap, joint planting with live stakes, brush layering, willow bundles, coconut fibre rolls, and erosion control blankets. The study's findings will also prove valuable in advanced simulations of channel migration, encompassing both lateral and vertical movements, and their repercussions on the adjacent river corridor. These simulations will aid in assessing the extent of land degradation and in formulating plans for effective riverbank protection and management measures. Lastly, the rationale of the study will have practical applications in predicting sediment yield, particularly for downstream rivers with tributaries that feed into a reservoir or impoundment. Sediment transport resulting from riverbank erosion and mass wasting due to bank failures can significantly affect the river's carrying capacity. Consequently, the quantification of sediment yield will enable the formulation of mitigation strategies aimed at maintaining an appropriate river carrying capacity, particularly during periods of high flows.

## 5 Computer code availability

• Name of code: NN-ARX Riverbank Erosion Rate Predictor
• Developers: Azlinda Saadon
• Contact details: School of Civil Engineering, College of Engineering, Universiti Teknologi MARA (UiTM), Shah Alam, Selangor, Malaysia; e-mail: azlindasaadon@uitm.edu.my
• Year first available: 2018
• Hardware required: NN-ARX Riverbank Erosion Rate Predictor was simulated on a computer with Intel(R) Core (TM) i7-1065G7 CPU @ 1.30 GHz 1.50 GHz, 64-bit operating system, x64-based processor, and 16 GB ram.
• Software required: NN-ARX Riverbank Erosion Rate Predictor was interpreted with MATLAB 2018 Neural Network Toolbox, Statistics and Machine Learning Toolbox.
• Program language: The code is written in MATLAB 2018 Neural Network Toolbox, Statistics and Machine Learning Toolbox.
• Code availability: The source code of NN-ARX Riverbank Erosion Rate Predictor can be downloaded from the following GitHub repository: https://github.com/azlindasaadon/NNARX

## Data availability statement

Data will be made available on request.

No additional information is available for this paper.

## CRediT authorship contribution statement

**Azlinda Saadon:** Writing – original draft, Validation, Investigation, Formal analysis, Data curation, Conceptualization. **Jazuri Abdullah:** Writing – review & editing, Writing – original draft, Supervision. **Ihsan Mohd Yassin:** Writing – review & editing, Validation, Software. **Nur Shazwani Muhammad:** Writing – review & editing, Supervision, Project administration. **Junaidah Ariffin:** Writing – review & editing, Supervision, Resources, Project administration, Funding acquisition.

## Declaration of competing interest

The authors declare that they have no known competing financial interests or personal relationships that could have appeared to influence the work reported in this paper.
